# Interactive training workshop to improve prostate mpMRI knowledge: results from the ESOR Nicholas Gourtsoyiannis teaching fellowship

**DOI:** 10.1186/s13244-023-01574-8

**Published:** 2024-01-25

**Authors:** Tristan Barrett, Kang-Lung Lee, Fredrik Illerstam, Henrik S. Thomsen, Kartik S. Jhaveri, Vibeke Løgager

**Affiliations:** 1 Department of Radiology, Cambridge University Hospitals NHS Foundation Trust, Addenbrooke’s Hospital, Cambridge, UK; 2 Department of Radiology, Taipei Veterans General Hospital, Taipei, Taiwan; 3 School of Medicine, National Yang Ming Chiao Tung University, Taipei, Taiwan; 4 Collective Minds Radiology AB, 182 33 Danderyd, Sweden; 5 Department of Radiology, Herlev Gentofte University Hospital, Herlev, Denmark; 6 Joint Department of Medical Imaging, University Health Network, Mount Sinai Hospital and Women’s College Hospital, University of Toronto, 610 University Ave, 3-957, Toronto, ON M5G 2M9 Canada

**Keywords:** Urogenital neoplasms, Prostatic neoplasms, Magnetic resonance imaging, Learning curve

## Abstract

**Purpose:**

Prostate MRI is established for the investigation of patients presenting with suspected early prostate cancer. Outcomes are dependent on both image quality and interpretation. This study assessed the impact of an educational intervention on participants’ theoretical knowledge of the technique.

**Methods:**

Eighty-one clinicians from two centers with varying experience in prostate MRI participated. Baseline knowledge was assessed with 10 written and image-based multiple-choice questions (MCQs) prior to a course including didactic lectures and hands-on interactive workshops on prostate MRI interpretation. Post-course, participants completed a second 10-question MCQ test, matched by format, themes, and difficulty, to assess for any improvement in knowledge and performance. Results were assessed using the Wilcoxon rank sum test, and the Wilcoxon signed-rank test for paired data.

**Results:**

Thirty-nine participants, including 25/49 (51.0%) and 14/32 (43.8%) at each center completed both assessments, with their results used for subsequent evaluation. Overall, there was a significant improvement from pre- (4.92 ± 2.41) to post-course scores (6.77 ± 1.46), *p* < 0.001 and at both Copenhagen (5.92 ± 2.25 to 7.36 ± 1.25) and Toronto (3.14 ± 1.51 to 5.71 ± 1.20); *p* = 0.005 and *p* = 0.002, respectively. Participants with no prostate MRI experience showed the greatest improvement (3.77 ± 1.97 to 6.18 ± 1.5, *p* < 0.001), followed by intermediate level (< 500 MRIs reported) experience (6.18 ± 1.99 to 7.46 ± 1.13, *p* = 0.058), then advanced (> 500 MRIs reported) experience (6.83 ± 2.48 to 7.67 ± 0.82, *p* = 0.339).

**Conclusions:**

A dedicated prostate MRI teaching course combining didactic lectures and hands-on workshops significantly improved short-term theoretical knowledge of the technique for clinicians with differing levels of experience.

**Critical relevance statement:**

A dedicated teaching course significantly improved theoretical knowledge of the technique particularly for clinicians with less reporting experience and a lower baseline knowledge. The multiple-choice questions format mapped improved performance and may be considered as part of future MRI certification initiatives.

**Key points:**

• Prostate MRI knowledge is important for image interpretation and optimizing acquisition sequences.

• A dedicated teaching course significantly improved theoretical knowledge of the technique.

• Improved performance was more apparent in clinicians with less reporting experience and a lower baseline knowledge.

**Graphical Abstract:**

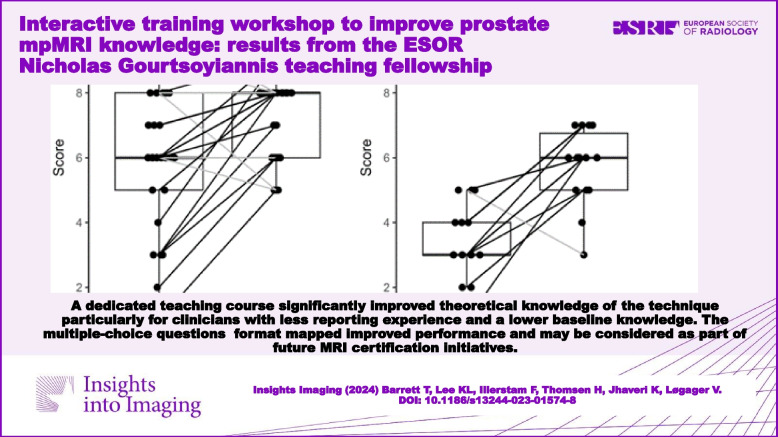

**Supplementary Information:**

The online version contains supplementary material available at 10.1186/s13244-023-01574-8.

## Introduction

Prostate cancer is the second commonest male cancer worldwide, with the incidence expected to increase by more than 50% by 2035 [1, 2]. MRI is now established as the initial diagnostic investigation of choice for patients presenting with suspected localized or locally advanced prostate cancer [3, 4], with improved outcomes demonstrated compared to the previous standard-of-care transrectal systematic biopsy [5–8]. The high demand for the technique necessitates that MRI be performed and reported in all healthcare settings.

The Prostate Imaging-Reporting and Data System (PI-RADS) guidelines aim to standardize both MRI acquisition and reporting [9], with patient-level outcomes being highly dependent on both image quality [10] and reader experience [11]. However, compliance to PI-RADS acquisition recommendations does not guarantee high quality [12, 13] and theoretical and practical knowledge are essential in order to adapt sequence parameters and ensure optimization [14, 15]. Moreover, despite the widespread adoption of the PI-RADS guidelines, there remains high inter-observer variability, even amongst expert readers [16, 17]. There is a known learning curve for prostate MRI interpretation [18], with clinical outcomes directly correlating to reader experience [19, 20], and with participation in dedicated training programmes being shown to improve reader performance [21–23].

The Nicholas Gourtsoyiannis Teaching Fellowship, established by the European School of Radiology (ESOR), is aimed at clinical and academic radiologists who wish to enhance their skills by delivering lectures and undertaking interactive workshops in a foreign environment. For the year 2022, the fellowship recipient (T.B.) is a subspecialist uro-radiologist who conducted prostate MRI teaching sessions at two separate centers in Denmark and Canada. The outcome of this dedicated training programme is presented, aiming to map the improvement in theoretical prostate MRI knowledge of participants from a variety of training backgrounds and with differing levels of experience.

## Methods

### Participants

The first teaching fellowship took place at Herlev Hospital, Copenhagen, Denmark, on August 22–23, 2022, and the second at Princess Margaret Cancer Center, Toronto, Canada, on November 9, 2022. In both centers, the teaching was offered in a hybrid format, with the majority attending in person and with online participants all from the same respective country. In total, 81 participated, with 49 in Copenhagen and 32 in Toronto.

### Hands-on workshop

Participants attended a prostate MRI workshop which included dedicated lectures and interactive cases, curated into themes based around the delivered talks. Workshops lasted up to 90 min, with access to the cases provided via a health technology company, Collective Minds™ (Stockholm, Sweden). Collective Minds™ provided a virtual classroom to host the lecturer's clinical prostate mpMRI cases, allowing course attendees to concurrently scroll and review studies. Participants were granted access to the cases 3 days before and 4 days after the workshops. Lectures were 10–15 min in length and covered core topics, including “Prostate MRI interpretation”, “Pitfalls in prostate MRI”, “Biparametric versus Multiparametric MRI” and “Quality Control within the prostate cancer diagnostic pathway” (Supplemental Data 1).

### Evaluation

Participants were asked to complete a brief pre-course questionnaire to record their current position and details of their prior prostate MRI experience. Within each group, there was mixed prostate MRI reporting experience, which was generally higher in the Copenhagen compared to the Toronto cohort (Table 1, Supplemental Data 2), with the latter course being mainly advertised to radiology fellows and residents.

**Table 1:** Baseline career and prostate MRI reader experience within the cohorts

	**All** **(*n* = 55)**	**Copenhagen** **(*n* = 30)**	**Toronto** **(*n* = 25)**
**Career experience**^**a**^			
Medical student	3 (5.5%)	0 (0%)	3 (12%)
Urology consultant	5 (10%)	5 (16.7%)	0 (0%)
Urology resident	1 (1.8%)	1 (3.3%)	0 (0%)
Radiology tesident (junior)	14 (25.5%)	4 (13.3%)	10 (40%)
Radiology resident (≥ year 4)	16 (29.1%)	4 (13.3%)	12 (48%)
Radiology consultant	16 (29.1%)	16 (53.3%)	0 (0%)
**Prostate MRI reading experience**^**a**^			
*None*	35 (63.6%)	12 (40%)	23 (92%)
*Beginner*	5 (9.1%)	3 (10%)	2 (8%)
*Intermediate*	7 (12.7%)	7 (23.2%)	0 (0%)
*Advanced*	8 (14.5%)	8 (26.7%)	0 (0%)

^a^Beginner: 1–100; intermediate: 100–500 mpMRI cases reported; advanced: > 500 mpMRI cases reported

In advance of the educational intervention, attendees were asked to take an assessment to evaluate their baseline prostate mpMRI knowledge. This assessment consisted of 10 multiple choice questions (MCQs), 6 written and 4 based on static prostate MR images (Supplemental data 3). In total 30/49 (61.2%) and 25/32 (78.1%) of participants completed the baseline assessment in Copenhagen and Toronto, respectively. Twenty-four hours after completion of the workshops a second assessment was made available to evaluate any improvement in knowledge and performance (Supplemental data 4). This was matched to the first in terms of number of written (6) and image-based (4) MCQs, difficulty level (easy = 2, intermediate = 5, hard = 3), and approximate themes. The test sets were curated by author T.B. in collaboration with a second author (V.L.). 25/49 (51.0%) and 14/32 (43.8%) of participants at each center completed both assessments, with their results used for subsequent evaluation. Participants who did not complete both pre- and post-course questionnaires were excluded from analysis, including one participant who only completed assessment 2.

The participants were categorized into three groups based on their experience level in independent prostate mpMRI reporting: beginner (with no prior experience in independent prostate mpMRI reporting), intermediate (with less than 500 mpMRI cases), and advanced (with more than 500 mpMRI cases).

### Statistical analysis

Statistical analysis was conducted by using R 4.2.2 (R Foundation for Statistical Computing, Vienna, Austria). The Wilcoxon rank sum test and Kruskal–Wallis test were performed for datasets with 2 and ≥ 3 groups, respectively. The Dunn test was used for post-hoc analyses when the results of the Kruskal–Wallis test reached significance. Paired data (i.e. pre- and post-course questionnaires from the same participant) were analyzed using the Wilcoxon signed-rank test. A significance level of *p* < 0.05 was considered statistically significant.

## Results

The pre-course assessment showed a mean score of 4.92 (± 2.41 SD) across 39 participants, with the post-course test demonstrating a significant improvement, with a mean score of 6.77 (± 1.46), *p* < 0.001 (Table 2, Supplemental data 5).

**Table 2:** Scores from pre- and post-course tests, reported as mean ± SD

	**Pre-course test**	**Post-course test**	***p*-value**
All (*n* = 39)	4.92 ± 2.41	6.77 ± 1.46	< 0.001
Copenhagen (*n* = 25)	5.92 ± 2.25	7.36 ± 1.25	0.005
Toronto (*n* = 14)	3.14 ± 1.51	5.71 ± 1.20	0.002

**Fig. 1:**
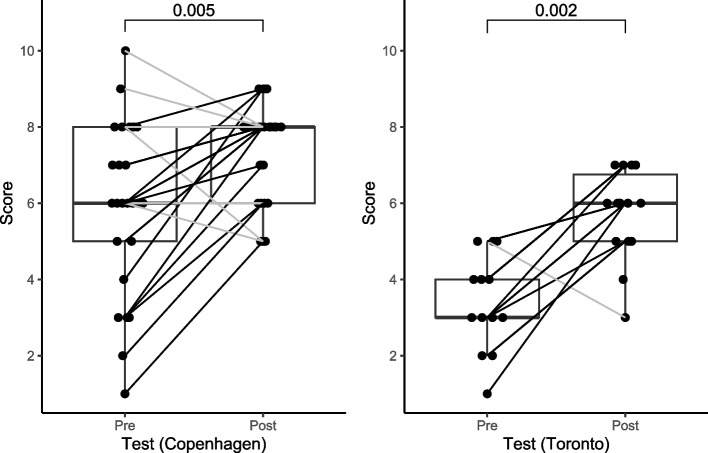
Box plot illustrating the distribution of scores from pre- and post-course tests for participants in each center

Amongst the participants from Copenhagen, the pre-course test revealed a mean score of 5.92 (± 2.25), which significantly increased to 7.36 (± 1.25) post-course, *p* = 0.005. For participants from Toronto, the mean pre-course score was 3.14 (± 1.51), and the mean post-course score 5.71 (± 1.20) *p* = 0.002, Table 2. Further analyses were performed to compare scores between the two cities. The pre-course scores from Copenhagen were significantly higher than Toronto (*p* < 0.001). Similarly, the post-course scores from Copenhagen were significantly higher than Toronto (*p* < 0.001). Significant improvements between the pre- and post-course scores were observed for both cities (Copenhagen: *p* = 0.005, Toronto: *p* = 0.002); Fig. 1. However, when directly comparing the post-course scores to the pre-course scores between Copenhagen and Toronto, the difference was not statistically significant (*p* = 0.090).

Separately, the pre- and post-course scores were analyzed based on participants’ experience levels. Significant differences were observed amongst experience groups (beginner, intermediate, and advanced) in both pre-test and post-test conditions (Supplemental Fig. 6). Pairwise comparisons revealed that, in pre-test scores, beginners scored significantly lower compared to both intermediate and advanced groups. This trend persisted in post-test scores. Amongst the 22 participants categorized as beginners, there was a significant increase in mean scores from 3.77 (± 1.97) at the pre-course assessment to 6.18 (± 1.5) at the post-course assessment, *p* < 0.001. The Intermediate experience group (*n* = 11) showed a moderate increase in the mean score from 6.18 (± 1.99) in the pre-course test to 7.46 (± 1.13) in the post-course test, but this did not reach statistical significance (*p* = 0.058). In the advanced group (*n* = 6), the participants demonstrated a slight but non-significant improvement in scores from the pre-course test (6.83 ± 2.48) to the post-course test (7.67 ± 0.82), *p* = 0.339 (Table 3).

**Table 3:** Scores from pre- and post-course tests categorized by participants’ experience, reported as mean ± SD

**Experience** ^a^	**Pre-course test**	**Post-course test**	***p*-value**
Beginner (*n* = 22)	3.77 ± 1.97	6.18 ± 1.5	< 0.001
Intermediate (*n* = 11)	6.18 ± 1.99	7.46 ± 1.13	0.058
Advanced (*n* = 6)	6.83 ± 2.48	7.67 ± 0.82	0.339

^a^Beginner: no independent prostate mpMRI reporting; intermediate: < 500 mpMRI cases reported; advanced: > 500 mpMRI cases reported

## Discussion

This study investigated the effect of a dedicated prostate MRI training programme incorporating didactic lectures and interactive cases, demonstrating a significant improvement in knowledge of the technique amongst clinicians from two separate healthcare systems, and with differing baseline levels of experiences. The findings highlight the effectiveness of the course in improving scores for all participants and within each center. While significant differences were observed between the centers in terms of pre-course and post-course scores, the impact did not significantly differ between the Copenhagen and Toronto cohorts.

Interpretation of prostate MRI is challenging, following a known learning curve; a recent ESUR consensus group suggested that > 1000 studies should be read to achieve the level of an expert [24]. It has previously been shown that dedicated teaching courses improve reader performance in MRI for both radiologists and urologists [21–23]. Formats have varied, but they typically include a mixture of didactic lectures and case-based teaching with feedback on image interpretation, delivered over periods as short as 2 days [23], or up to 20 weeks [21].

Certification has been proposed as a means of benchmarking the requirements for independent prostate MRI reporting [25–28]. Such a qualification could be multifaceted, incorporating a logbook of cases, peer-learning, course participation, continuing medical education credits, and pathology feedback [26]. Some of these components were included within the current training programme. A potential examination format has been proposed as either an online MRI case-based interpretation or a mixture of written and image-based multiple choice questions [26]. We tested the latter and importantly matched the question sets in terms of topic, format, and difficulty, to help map improvement in theoretical knowledge following educational workshops. The significant differences between the score for experience levels pre- and post-course imply that such testing may help to differentiate knowledge as part of a certification process; however, we aimed to evaluate learning, and it should be noted that certification itself does not directly equate to knowledge and experience, nor indeed to competence. Although the group mean scores (out of 10) may appear modest, even after the teaching intervention (7.36 at Copenhagen and 5.71 at Toronto, respectively), the questions were deliberately set to be challenging to avoid a “ceiling effect” where resultant high scores on the baseline questions would limit the scope to map any improvement. It was notable that the Toronto cohort scored lower overall on baseline testing, likely reflect the relatively lower a priori prostate MRI reading experience within this group. Notably, there was a statistical difference between beginner versus the intermediate and experience level groups at both baseline and post-course, implying that such testing may be able to differentiate candidate knowledge. It was also apparent that readers with beginner-level experience showed a larger and statistically significant increase in scoring compared to those with intermediate or advanced-level experience (which did not reach significance), suggesting a greater scope for improvement in performance, however, this would need to be confirmed by further testing in more closely matched cohort studies.

This study has several limitations, including the relatively small number of participants and the relatively low number of multiple-choice questions assessed, to balance the breadth of knowledge covered and the time burden for completion. Not all attendees completed both the baseline and post-course testing, which may have led to a selection bias within and between centers, however, the response rate was generally comparable to other studies in the field [29, 30] and was overall relatively similar between the two centers (51.0% and 43.8%). The question sets were matched for topic and difficulty, however, there remains potential for mismatch and randomization of the assessment order between candidates may have been more appropriate. Participants were not tested on full prostate mpMRI datasets, however, the format employed has been suggested as a practical approach for examinations within prospective certification programmes [26], and this pilot shows the potential for the MCQ format to map improvements in theoretical prostate MRI knowledge. Finally, the post-course questions were available for completion shortly after the applied learning event, and no follow-up testing was performed to evaluate for longer-term retained knowledge; this is an area for future work.

In conclusion, this study shows that a dedicated prostate mpMRI teaching course combining didactic lectures and practical workshops can improve the theoretical knowledge of the technique for clinicians with differing levels of experience, and provides evidence that a multiple-choice question format can be employed as an assessment tool for this purpose.

### Supplementary Information

**Additional file 1.**

## Data Availability

Further information is available from the corresponding author on reasonable request.

## Abbreviations

ESOR: European School of Radiology
ESUR: European Society of Urogenital Radiology
MCQ: Multiple-choice questions
mpMRI: Multiparametric magnetic resonance imaging
PI-RADS: Prostate Imaging-Reporting and Data System
