# Supplementary material for: Interactive training workshop to improve prostate mpMRI knowledge: results from the ESOR Nicholas Gourtsoyiannis teaching fellowship
Source: Insights Imaging. 2024 Jan 25;15:27. doi: 10.1186/s13244-023-01574-8 (PMC10810764; doi:10.1186/s13244-023-01574-8)
Supplement: Supplementary file 1 — Additional file 1. [file 13244_2023_1574_MOESM1_ESM.docx]

**Interactive training workshop to improve prostate mpMRI knowledge: results from the ESOR Nicholas Gourtsoyiannis teaching fellowship**

**ELECTRONIC SUPPLEMENTARY MATERIAL**

**Supplemental Data 1: Educational content, Copenhagen**

**Monday 22^nd^ August, 2022 (15:30 - 18:30)**

15:30 - 15:45 Lecture: “Prostate MRI interpretation”
15:45 - 17:00 Hands-on interactive workshop cases*
17:00 - 17:15 Lecture: “Biparametric versus Multiparametric MRI”
17:15 - 18:30 Hands-on interactive workshop cases*

**Tuesday 23^rd^ August, 2022 (15:30-19:15)**
15:30 - 15:45 Lecture: “Pitfalls in prostate MRI”
15:45 - 17:00 Hands-on interactive workshop cases*
17:00 - 17:15 Lecture: “Quality Control within the prostate cancer diagnostic pathway”
17:15 - 18:30 Hands-on interactive workshop cases*
18:30 - 19:15 Lecture: “Certification in Prostate MRI: The next step for ensuring quality”

** = Theme-based interactive workshop, up to 9 cases in each*

**Supplemental Data 2**

**Pre-Course Written Multiple Choice Questions (1-6)

1. What is the current version of the PI-RADS international guidelines for prostate MRI reporting?**
(a) PI-RADS version 1
(b) PI-RADS version 1.2
(c) PI-RADS version 2
(d) PI-RADS version 2.1
(e) PI-RADS version 2.2
(f) PI-RADS version 3

**[Easy]
Answer: (d) PI-RADS version 2.1**

**2. How do you PI-RADS score the PZ** **if DWI fails and is technically inadequate?**(a) DCE becomes dominant
(b) Limit assessment to staging only
(c) Perform MR spectroscopy
(d) Proceed straight to biopsy
(e) T2WI becomes dominant
 **[Hard]
Answer: (e) T2WI becomes dominant**Although DCE is the secondary sequence in the PZ, it is only scored out of 2 (positive/negative). T2WI is the tertiary sequence in the PZ, but becomes dominant in this case, bypassing DCE, to enable a PI-RADS score out of 5. *“Limit assessment to staging only”* is reserved for MRI cases where NO functional sequence is available.

**3. You score a lesion “PI-RADS 2” nodule on T2WI in the TZ. What is required to up-score this to a “3”?**
(a) DCE positive
(b) DWI score 4 or 5
(c) DWI score 5
(d) DWI score 4 or 5 and DCE positive
(e) DWI score 5 and DCE positive

**[Hard]
Answer: (b) DWI score 4 or 5**The secondary sequence in the TZ is DWI (assuming technically adequate). There is a slight quirk in the scoring system in that a DWI score of 4 (note 5) is required to up-score a T2WI score of 2 to 3 (“2+1) whereas a score 5 (not 4) is required in the TZ to convert T2WI score 3 to 4 (“3+1”), thus as well as being high probability on DWI it needs to be at least 15 mm in size or demonstrating features of ECE in order to up-score.

**4. In PI-RADS version 2.1, what is the recommended high b-value DWI sequence to use for calculation of an ADC map?**(a) 500
(b) 750
(c) 800-1000
(d) ≥1400
(e) ≥2000
 **[Intermediate]
Answer: (c) 800-1000**PI-RADS v2.1 states that a minimum of 2 b-values are required to calculate an ADC map, and that the “maximum b-value used to calculate ADC is recommended to be ≤1,000 sec/mm2 to avoid diffusion kurtosis effects that have been described at higher b-values.”

**5. How do you define local (T) staging on MRI?**

(a) DWI is the key sequence for staging of PZ lesions

(b) DWI is the key sequence for staging of PZ and TZ lesions

(c) DWI is the key sequence for staging of PZ lesions and T2 is key for TZ lesions

(d) T2W is the key sequence for staging of PZ and TZ lesions

(e) T2W is the key sequence for staging of TZ lesions

**[Intermediate]**

**Answer: (d) T2W is the key sequence for staging of PZ and TZ lesions**

High spatial resolution T2W imaging is essential for accurate assessment of extracapsular extension (ECE) and seminal vesicle invasion regardless of the lesion location.

**6. How should you assess for locally recurrent disease in patients post prostatectomy or post radiotherapy?**(a) PERCIST
(b) PI-RADS
(c) PI-RR
(d) RECIST
(e) PRECISE

**[Intermediate]
Answer: (c) PI-RR**
The Prostate Magnetic Resonance Imaging for Local Recurrence Reporting (PI-RR) guidelines were published in 2021 (Panebianco V, et al. Eur Urol Oncol. 2021; 4(6):868-876. doi: 10.1016/j.euo.2021.01.003)

**Pre-Course Image-based Questions (7-10)

7. 56-year-old man with an elevated PSA of 7.1 ng/mL.


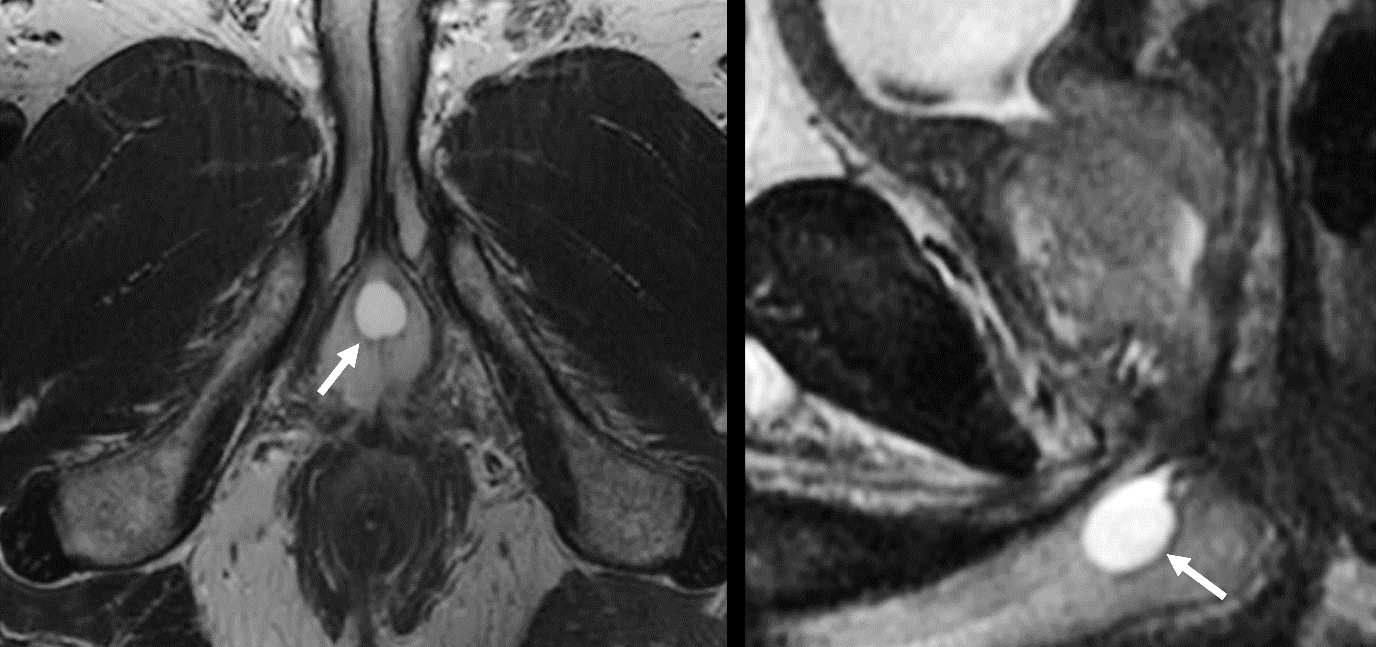
**

**What is the structure/lesion (arrow) on these T2W images?**

(a) Cowper’s duct cyst

(b) Cystic BPH nodule

(c) Ejaculatory duct cyst

(d) Mullerian duct cyst

(e) Utricle cyst

**[Intermediate]**

**Answer: (e) Cowper’s duct cyst**

Cowper’s duct cysts consists of cystic dilatation of Cowper’s gland ducts. The bulbourethral glands (Cowper glands) are paired paraurethral glands located in the urogenital diaphragm near the bulbous urethra. A retention cyst can occur with obstruction of the duct and gland, causing variable appearances of Cowper’s ducts and at their insertion into the bulbous urethra.

**8. On this T2-weighted coronal image, what does the labelled anatomical structure represent.**


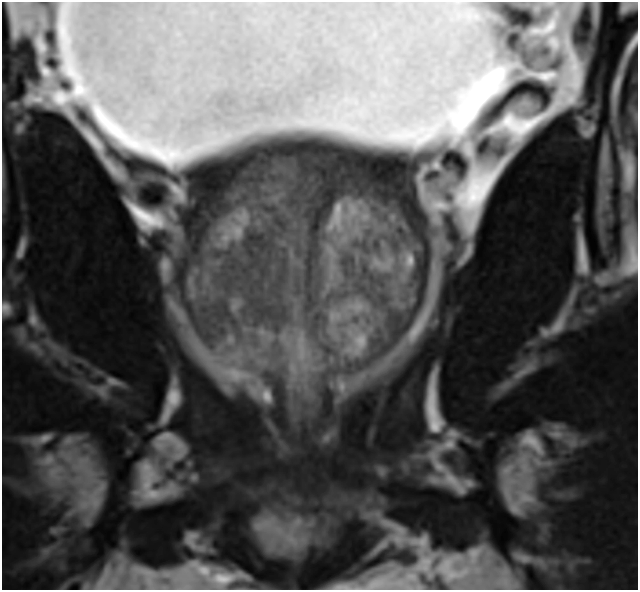


(a) Surgical capsule at the interface between the peripheral and transitional zones

(b) Levator ani muscle

(c) External sphincter complex

(d) Verumontanum

(e) Prostatic urethra

**[Intermediate]**

**Answer: (c) External sphincter complex**

**9. 57-year-old man. PSA 9.8 ng/ml. Biopsy naïve.**

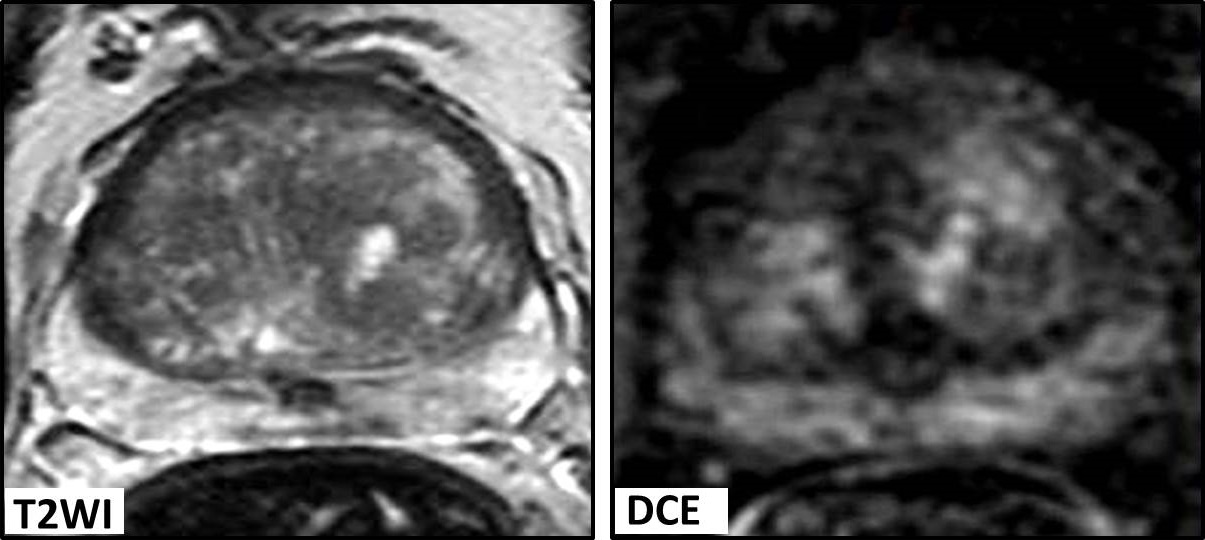

 **Regarding scoring of the right PZ area?**(a) T2 ≥PI-RADS 3 and DCE Positive
(b) T2 ≥PI-RADS 3 and DCE Negative
(c) T2 Negative and DCE Positive
(d) T2 Negative and DCE Negative
 **Answer: (d) T2 Negative and DCE Negative**
For DCE to be positive there need to be 3 criteria met: focal and early enhancement (vs normal) and matches a suspicious area on T2/DWI. In this case, there is diffuse enhancement and no T2 abnormality. In this case, there is prostatitis, which may be a useful finding to report as this may explain the raised PSA, and biopsy may be more likely to result in sepsis.

**[Hard]**

**Supplemental Data 3**

**Post-Course Written Multiple Choice Questions (1-6)

1. Which of the following does not form a part of McNeal’s anatomical divisions of the prostate, adopted by PI-RADS?**
(a) Anterior fibromuscular stroma
(b) Central gland
(c) Peripheral zone
(d) Transition zone

**[Hard]**
**Answer: (b) Central gland**
McNeal's scheme divides the adult human prostate into an anterior fibromuscular zone and three glandular zones (the central zone surrounds the ejaculatory ducts, the transition zone surrounds the urethra, and the peripheral zone surrounds both). McNeal J.E. The zonal anatomy of the prostate. Prostate. 1981; 2: 35-49.

**2. How should you assess for change in follow-up active surveillance studies?**(a) PI-RADS version 1
(b) PI-RADS version 2.1
(c) PERCIST
(d) RECIST
(e) PRECISE

**[Intermediate]
Answer: (e) PRECISE**The PRECISE guidelines have been proposed as a scoring system for MRI progression on AS (Moore, et al. Eur Urol. 2017;71(4):648-655). Herein:
1= Resolution of previous features suspicious on MRI
2= Reduction in volume and/or conspicuity of previous features suspicious on MR
3 = Stable MRI appearance: no new focal/diffuse lesions
4 = Significant increase in size and/or conspicuity of features suspicious for prostate cancer
5 = Definitive radiologic stage progression

**3. You score a lesion as “PI-RADS 3” on T2WI in the TZ. What is required to up-score this to a “4”?**
(a) DCE positive
(b) DWI score 4 or 5
(c) DWI score 5
(d) DWI score 4 or 5 and DCE positive
(e) DWI score 5 and DCE positive

**[Hard]
Answer: (c) DWI score 5**The secondary sequence in the TZ is DWI (assuming technically adequate). There is a slight quirk in the scoring system in that a DWI score of 5 (not 4) is required in the TZ to convert T2WI score 3 to 4, thus as well as being high probability on DWI it needs to be at least 15 mm in size or demonstrating features of ECE in order to up-score.

**4. In PI-RADS version 2.1, what is the recommended high b-value DWI sequence to perform if SNR permits?**(a) 500
(b) 750
(c) 800-1000
(d) ≥1400
(e) ≥2000
 **[Easy]
Answer: (d) ≥1400**PI-RADS v2 states that “there is no currently widely accepted optimal ‘high b-value’, but if adequate SNR permits, b-values of 1400-2000 sec/mm2 or higher seem to be advantageous”. For calculation of ADC maps “…the lowest b-value should be set at 50-100 sec/mm^2^ and the highest should be 800-1000 sec/mm^2^”.

**5. What is the definition of a PIRADS 5 lesion?**

(a) Lesion showing high signal on DWI

(b) Lesion size ≥ 15 mm

(c) Lesion size ≥ 15 mm and/or showing invasive features

(d) Marked restricted diffusion with ADC values < 800 ×10^-3^ mm^2^/s

(e) Type 3 DCE curve

**[Intermediate]**

**Answer: (c) Lesion size ≥ 15 mm and/or showing invasive features**

PIRADS 5 lesion is defined by the lesion size > 15 mm OR by showing invasive features.

**6. What assessment scale would you apply to evaluate image quality when interpreting a prostate mpMRI?**(a) Likert
(b) PI-RADS
(c) PI-QUAL
(d) PI-RR
(e) PRECISE

**[Intermediate]
Answer: (c) PI-QUAL**
The Prostate Imaging Quality (PI-QUAL) scoring system was developed from 22 centres within the PRECISION trial and was published in 2020 (Eur Urol Oncol. 2020; 3:615-19).

**Post-Course Image-based Questions (7-10)

7.** **64 year old man, persistently elevated PSA at 11.5 ng/mL.**

**
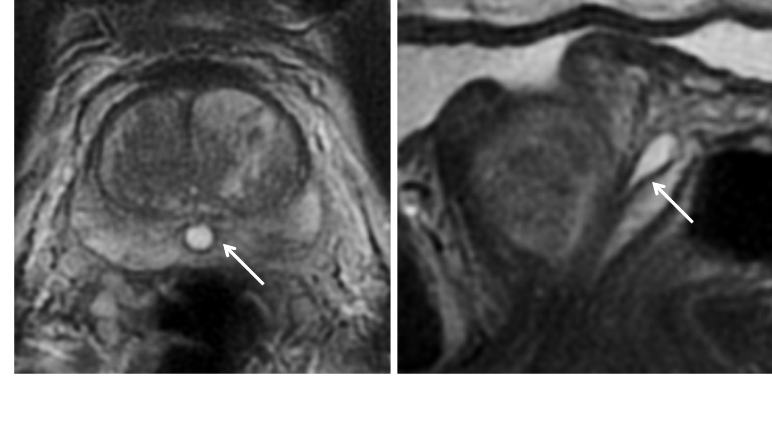
**

**What is the structure/lesion (arrow) on these T2W images?**

(a) Abscess in the ejaculatory duct

(b) Cystic BPH nodule

(c) Ejaculatory duct cyst

(d) Mullerian duct cyst

(e) Utricle cyst

**[Easy]**

**Answer: (e) Utricle cyst**

Prostatic utricle cysts by definition arise from the level of the [verumontanum](https://radiopaedia.org/articles/verumontanum) and are in the midline. They tend to be pear-shaped and as opposed to Mullerian duct cysts do not extend beyond the base of the prostate.

**8. Axial T2-weighted MR images in different biopsy naïve men.**
**
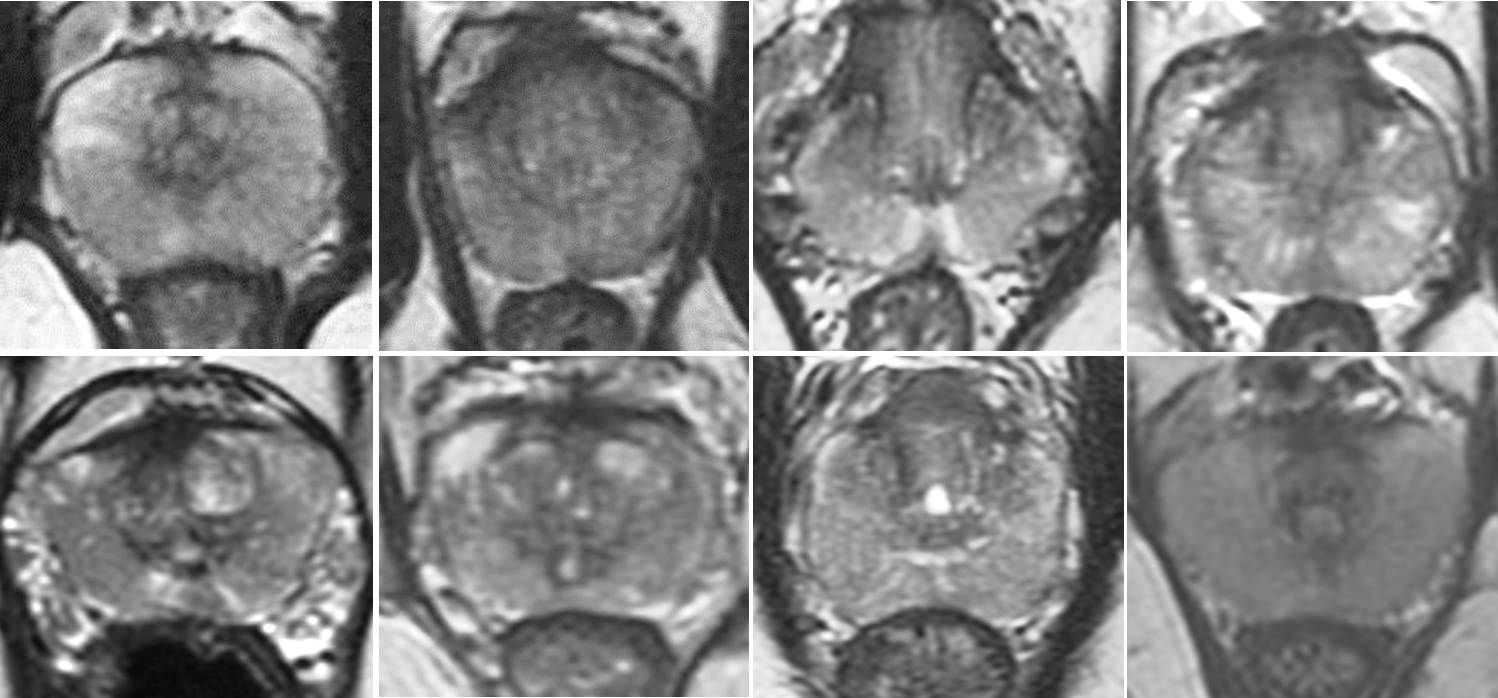
**
**What is the most likely age-range of the patients?**(a) <55 years old
(b) 55 - 65 years old
(c) 65 - 75 years old
(d) >75 years old

**[Intermediate]
Answer: (a) <55 years old**
The “normal” homogeneous high signal intensity seen in the PZ on T2WI relates to older patients with simple atrophy or cystic-type atrophy. Younger patients typically have a mixture of normal glands and partial/simple atrophy (Bura V, Barrett T. Eur Radiol. 2021; 31(7):4908-4917). The gland will typically appear on T2WI as isointense and ill-defined, on DWI as isointense on high-b-value images with intermediate ADC, and on DCE showing moderately contrast enhancing with linear or plateau-type curve. Also of note in these cases is the (young age-appropriate) relatively minimal volume TZ.

**9. For this bpMRI case, how would you assess for lesions using the PI-RADS system?


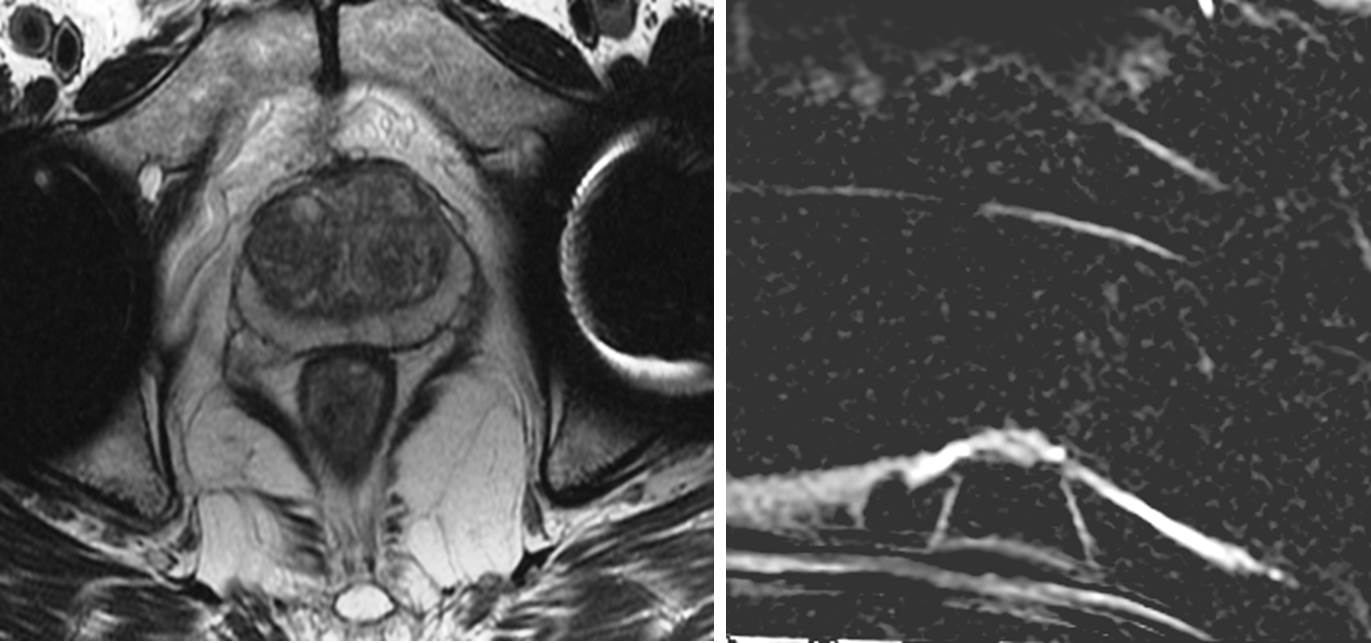
**(a) Advise to proceed straight to biopsy
(b) Limit assessment to staging
(c) T1WI can help for lesion detection
(d) The study is automatically PI-RADS 3

**[Hard]**

**Answer: (b) Limit assessment to staging**

**10. What does likely this represent?**
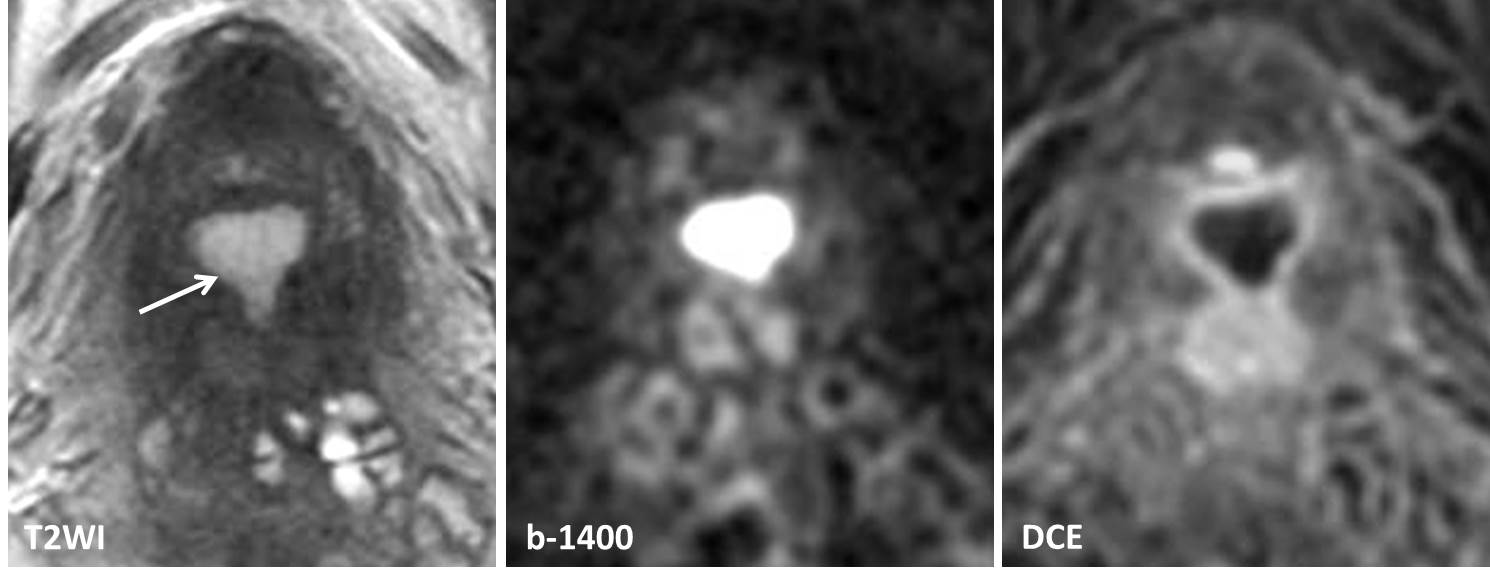


(a) Abscess
(b) Post TURP appearance
(c) Tumour
(d) Utricle cyst
 **[Intermediate]**

**Answer: (a) Abscess**

NB marked restricted diffusion (TURP cavity will not have). Both can have rim enhancement at DCE


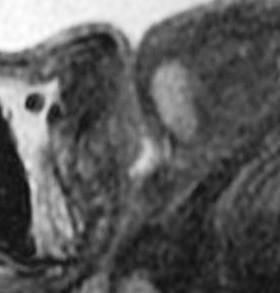

Sagittal image confirms no connection to bladder neck

**10. 65-year-old, PSA 6.04**


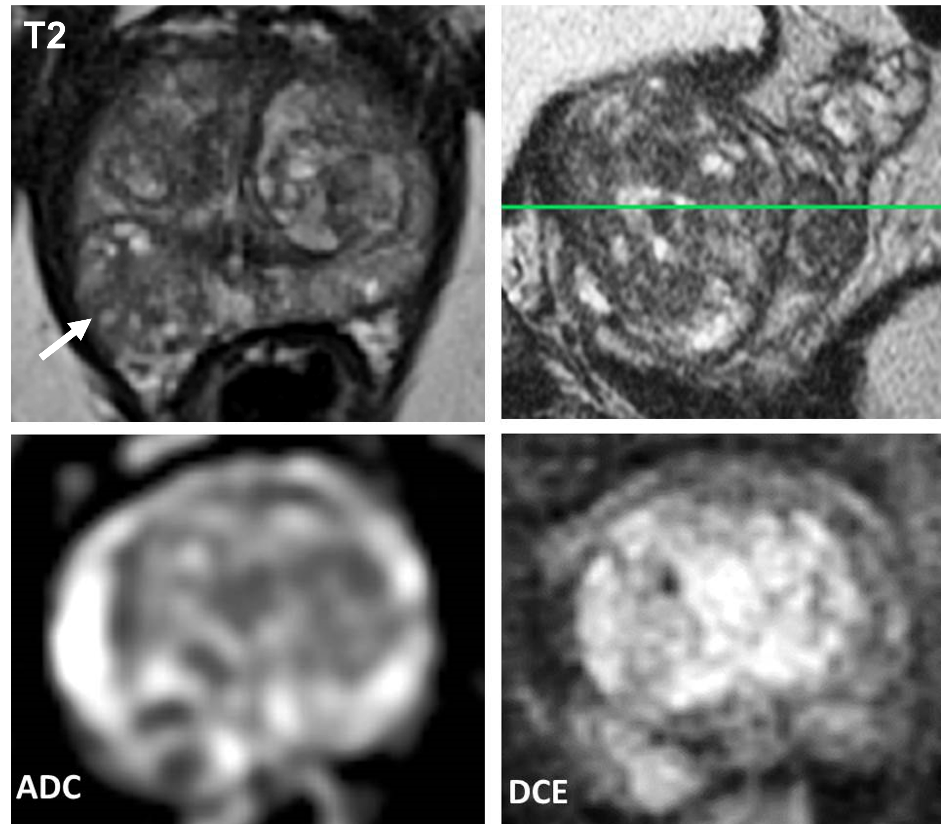


**What is the most likely diagnosis?**(a) Granulomatous prostatitis

(b) Left PZ tumour with early ECE

(c) Extruded BPH nodule

(d) Metastatic lesion

Answer: (c)

**[Easy]**

**Supplemental Data 4**

|  | | **All  (n = 39)** | **Copenhagen  (n = 25)** | **Toronto  (n = 14)** |
| --- | --- | --- | --- | --- |
| **Career Experience** | | | | |
| Medical Student | | 0 (0%) | 0 (0%) | 0 (0%) |
| Urology Consultant | | 3 (10%) | 3 (16.7%) | 0 (0%) |
| Urology Resident | | 1 (1.8%) | 1 (3.3%) | 0 (0%) |
| Radiology Resident (Junior) | | 11 (25.5%) | 4 (13.3%) | 7 (50%) |
| Radiology Resident (≥Year 4) | | 9 (29.1%) | 2 (13.3%) | 7 (50%) |
| Radiology Consultant | | 15 (29.1%) | 15 (53.3%) | 0 (0%) |
| **Prostate MRI Reading Experience *** | | | | |
| *None* | 22 (56.4%) | | 9 (36%) | 13 (92.9%) |
| *Beginner* | 4 (10.3%) | | 3 (12%) | 1 (7.1%) |
| *Intermediate* | 7 (17.9%) | | 7 (28%) | 0 (0%) |
| *Advanced* | 6 (15.4%) | | 6 (24%) | 0 (0%) |

**Table.** Baseline career and prostate MRI reader experience within the cohorts for the 39 attendees completing the pre and post course assessments. *Beginner: 1-100; Intermediate: 100-500 mpMRI cases reported; Advanced: > 500 mpMRI cases reported.

**Supplemental Data 5**

| **#** | **Career Experience** | **Prostate MRI** | **Pre** | **Post** | **Change** |
| --- | --- | --- | --- | --- | --- |
|  |  | **Experience *** |  |  |  |
| T1 | Radiology Resident (Junior) | None | 3 | 6 | 3 |
| T2 | Radiology Resident (Junior) ⱡ | None | 5 | 6 | 1 |
| T3 | Radiology Resident (>Y4) | None | 3 | 7 | 4 |
| T4 | Radiology Resident (>Y4) | None | 4 | 7 | 3 |
| T5 | Radiology Resident (>Y4) | None | 2 | 5 | 3 |
| T6 | Radiology Resident (>Y4) | None | 5 | 6 | 1 |
| T7 | Radiology Resident (Junior) | None | 0 | 4 | 4 |
| T8 | Radiology Resident (>Y4) ⱡ | Beginner | 4 | 7 | 3 |
| T9 | Radiology Resident (>Y4) | None | 3 | 6 | 3 |
| T10 | Radiology Resident (Junior) | None | 4 | 7 | 3 |
| T11 | Radiology Resident (>Y4) | None | 1 | 6 | 5 |
| T12 | Radiology Resident (Junior) | None | 5 | 3 | -2 |
| T13 | Radiology Resident (Junior) | None | 2 | 5 | 3 |
| T14 | Radiology Resident (Junior) | None | 3 | 5 | 2 |
| C1 | Radiology Consultant | Intermediate | 8 | 8 | 0 |
| C2 | Radiology Consultant ⱡ | Intermediate | 7 | 8 | 1 |
| C3 | Urology consultant | None | 2 | 6 | 4 |
| C4 | Radiology Resident (>Y4) ⱡ | Advanced | 6 | 8 | 2 |
| C5 | Radiology Consultant | Intermediate | 8 | 8 | 0 |
| C6 | Radiology Resident (Junior) | None | 6 | 7 | 1 |
| C7 | Radiology Consultant | None | 5 | 8 | 3 |
| C8 | Urology consultant | None | 6 | 9 | 3 |
| C9 | Radiology Resident (Junior) | None | 8 | 5 | -3 |
| C10 | Radiology Consultant | Advanced | 9 | 8 | -1 |
| C11 | Radiology Consultant | Beginner | 6 | 5 | -1 |
| C12 | Radiology Consultant | Intermediate | 8 | 9 | 1 |
| C13 | Radiology Resident (>Y4) | Beginner | 3 | 8 | 5 |
| C14 | Urology consultant ⱡ | Advanced | 10 | 8 | -2 |
| C15 | Radiology Consultant | Intermediate | 6 | 6 | 0 |
| C16 | Radiology Consultant ⱡ | Beginner | 3 | 7 | 4 |
| C17 | Radiology Resident (Junior) | None | 1 | 5 | 4 |
| C18 | Radiology Consultant | Advanced | 7 | 8 | 1 |
| C19 | Radiology Consultant | Intermediate | 8 | 8 | 0 |
| C20 | Radiology Consultant | Advanced | 6 | 8 | 2 |
| C21 | Radiology Consultant | None | 6 | 6 | 0 |
| C22 | Radiology Resident (Junior) | None | 5 | 8 | 3 |
| C23 | Urology Resident | None | 4 | 9 | 5 |
| C24 | Radiology Consultant | Intermediate | 7 | 8 | 1 |
| C25 | Radiology Consultant | Advanced | 3 | 6 | 3 |

**Table.** Career and prostate MRI reader experience with individual performance in pre- and post-teaching assessments. T = Toronto, C = Copenhagen. ⱡ = attended online. *Beginner: 1-100; Intermediate: 100-500 mpMRI cases reported; Advanced: > 500 mpMRI cases reported.

**Supplemental Figure 6.** Box plot illustrating the differences in scores based on experience level for pre- and post-course tests.

**
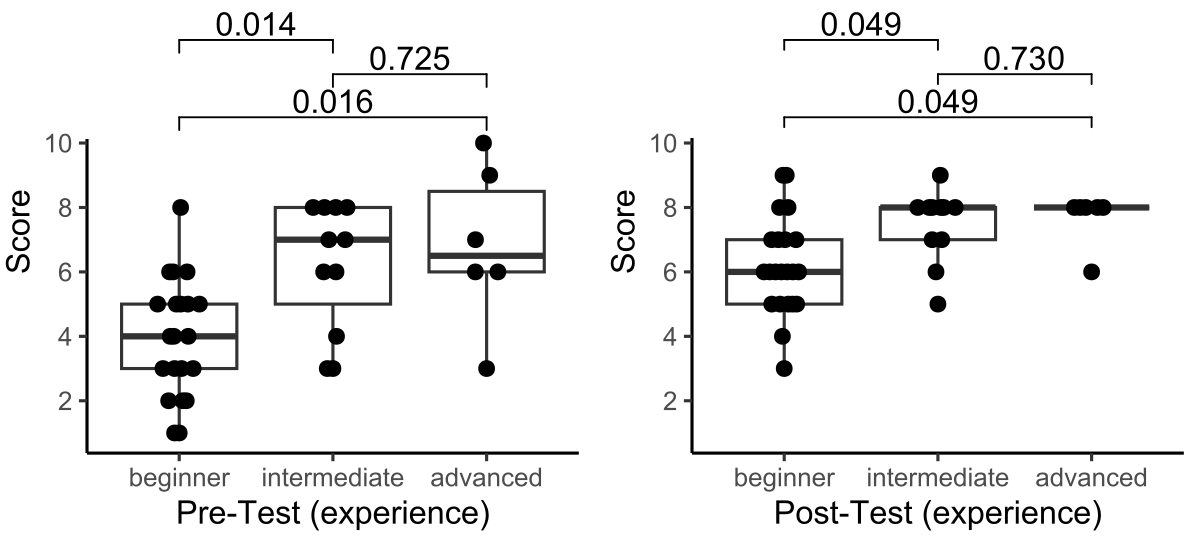
**
